# Genetic and Molecular Mechanisms of Detoxification and Immunity in Honeybees (*Apis mellifera*)

**DOI:** 10.3390/insects17060559

**Published:** 2026-05-28

**Authors:** Zunair Ahsan, Faouzi Haouala, Usama Abdullah, Umar Sajid Kayani, Mokhtar Rejili

**Affiliations:** 1 College of Animal Science and Technology, Yangzhou University, 88 South University Rd, Yangzhou 225009, China; mh24057@stu.yzu.edu.cn (Z.A.); usama.kpr124@gmail.com (U.A.); umarkiyani7171@gmail.com (U.S.K.); 2 Department of Biology, College of Sciences, Imam Mohammad Ibn Saud Islamic University (IMSIU), Riyadh 11623, Saudi Arabia; fmhaouala@imamu.edu.sa

**Keywords:** *Apis mellifera*, detoxification, innate immunity, social immunity, multi-stressor interactions, gut microbiota, oxidative stress, colony resilience

## Abstract

Honeybee (*Apis mellifera*) health is governed by the integrated action of detoxification, immunity, and microbiota within complex environmental contexts. The coordinated detoxification system (DETOXome), primarily active in the midgut, fat body, and Malpighian tubules, includes cytochrome P450s, glutathione S transferases, carboxylesterases, and ABC transporters, and functions in concert with innate immune pathways such as Toll, Imd, Jak/STAT, JNK, antimicrobial peptides, and RNA interference. Cellular maintenance mechanisms, including heat shock proteins, proteostasis, and antioxidant defenses, support these systems under chemical, thermal, and pathogen-induced stress. Multi-stressor exposures encompassing pesticides, pathogens, nutritional limitations, and climate variations interact to affect physiological resilience, behavior, and colony function. This review synthesizes molecular, organ-specific, and colony-level evidence to provide a mechanistic framework connecting environmental stressors to detoxification and immune responses. Predictive markers derived from transcriptomic, proteomic, and microbiome analyses offer early detection of sublethal stress, while genomic and selective breeding strategies hold the potential to enhance honeybee resilience. By integrating stress pathways across biological scales, this review advances a unified model of honeybee health that moves beyond descriptive lists to highlight cross-system interactions driving colony survival.

## Simple Summary

The fat body, midgut, and Malpighian tubules coordinate the integration of gut microbiota, innate immunity, and detoxification pathways in honeybee health. Rather than functioning independently, these molecular systems are interconnected and supported by behavioral defenses at the colony level, forming a multi-layered response to environmental stressors. However, the combined pressures of infections, pesticide exposure, and nutritional limitations can gradually compromise this resilience. Even sublethal disruptions at the molecular level may lead to behavioral changes and reductions in colony performance. These findings highlight the importance of considering coordinated physiological and ecological mechanisms in understanding honeybee health under contemporary environmental challenges.

## 1. Introduction

Despite substantial research on honeybee detoxification and immunity separately, a comprehensive synthesis integrating these systems within the context of multi-stressor exposures remains lacking. Existing reviews often focus on individual stressors, gene families, or immune pathways without explicitly linking molecular mechanisms to ecological and colony-level outcomes. This review addresses this gap by providing a systems-level perspective that combines detoxification genes (DETOXome), immune signaling pathways, organ-specific roles, gut microbiota interactions, and the effects of environmental stressors, offering a unified framework to understand honeybee resilience. It differs from previous work by emphasizing cross-scale integration and highlighting multi-stressor implications for colony health and survival. In honeybees, detoxification is mainly controlled by a coordinated gene network called the DETOXome. This includes cytochrome P450 monooxygenases (CYPs), carboxylesterases (COEs), glutathione S-transferases (GSTs), ABC transporters, and UDP-glycosyltransferases. A comprehensive analysis spanning 47 transcriptomes found that over 50% of detoxification genes are activated simultaneously, especially during larval and adult stages. These detoxification genes are primarily expressed in the fat body, midgut, and Malpighian tubules, suggesting that these tissues play a crucial role in the metabolism of environmental pollutants and pesticides [1]. Honeybee defense operates at two interconnected but distinct levels. At the individual level, innate immunity includes cellular defenses, such as hemocyte-mediated encapsulation and phagocytosis, and humoral defenses, including antimicrobial peptides such as defensins, apidaecin, abaecin, and hymenoptaecin [2]. At the colony level, social immunity refers to collective behavioral defenses, including hygienic behavior, social segregation, removal of infected individuals, and allogrooming, which reduce pathogen transmission within the colony [3,4]. These colony-level behaviors protect colony health by lowering pathogen exposure and transmission rather than directly enhancing innate immune mechanisms in individual bees. Furthermore, the gut microbiota contributes to both detoxification and immune regulation. Beneficial bacteria such as *Bartonella apis* and *Apilactobacillus kunkeei* can modulate Toll and Imd signaling pathways and increase antimicrobial peptide expression, thereby influencing host immune responses [5]. An age-related transition from structural to molecular defense is suggested by the higher levels of cuticle melanization and AMP expression seen in older foragers [6]. By mediating eicosanoid signaling in response to bacterial infections, the enzyme phospholipase A2 (PLA2) also plays a crucial role in humoral and cellular immune responses [7].

## 2. Environmental Stressors and Exposure

Honeybee colony health is shaped by the combined effects of pesticide exposure, pathogen pressure, and nutritional status. Chronic exposure to low-dose pesticide mixtures can interact with infections and poor nutrition to impair immunity, behavior, and overall colony resilience [8,9,10,11,12]. Nutritional quality, including the amino acid and phytochemical composition of pollen and nectar, influences immune competence and detoxification capacity, making nutritionally stressed colonies more vulnerable to chemical and pathogen stress [13,14,15].

Climate variability further influences these interactions by altering floral availability, pesticide dynamics, and parasite pressures, which indirectly affect colony resilience. Honeybees encounter systemic pesticides through multiple environmental pathways, including contaminated pollen and nectar, which can extend exposure to all colony members and result in sublethal effects, reinforcing the need to consider environmental mixtures rather than single compounds [16,17].

Honeybees (*Apis mellifera*) express heat shock proteins (HSPs) as a central cellular defense mechanism against thermal and environmental stress. Acting as molecular chaperones, HSPs stabilize and refold damaged proteins, maintain proteostasis, and preserve cellular function under adverse conditions. Multiple HSP families, including sHSPs, HSP40, HSP60, HSP70, and HSP90, are induced in response to temperature extremes, pollutants, and pathogen exposure, demonstrating their role in broad stress resilience [18]. In particular, DnaJ/HSP40 proteins are strongly upregulated during heat stress and contribute to survival by enhancing antioxidant defenses and limiting oxidative damage [19]. Variation in the expression of major HSP genes such as HSP70, HSP90, and HSC70 has also been associated with differences in thermal tolerance, suggesting a role in phenotypic plasticity under changing environmental conditions [20]. Beyond thermal stress, HSPs interact with antioxidant and immune pathways to support cellular homeostasis during chemical and pathogen exposure, thereby strengthening overall resilience to combined environmental stressors [21]. This multifunctional support role enables detoxification enzymes and immune effectors to maintain activity under stress and contributes to organismal resilience to environmental stressors, enhancing overall homeostatic capacity under combined challenges. Proteostasis, defined by the coordinated regulation of protein synthesis, folding, and degradation, further contributes to cellular homeostasis and resilience by preventing the accumulation of misfolded proteins that can compromise both detoxification enzymes and immune effectors [22]. The integration of these cellular maintenance systems with traditional detoxification and immune pathways ensures functional integrity under multi-stressor exposures, reinforcing their central role in honeybee environmental stress resilience. Cellular maintenance mechanisms, encompassing heat shock proteins (HSPs), antioxidant defenses, and proteostasis networks, act as a transversal support layer that underpins both detoxification and immune processes in honeybees facing environmental stressors. In *Apis mellifera*, HSPs and associated chaperone systems preserve protein structure and function under diverse stress conditions, buffering the effects of chemical-, thermal-, and pathogen-induced perturbations by stabilizing cellular proteins and interacting with antioxidant pathways to maintain redox balance [21]. Honeybees (*Apis mellifera*) exhibit pronounced physiological and immunological responses to elevated temperatures, demonstrating that heat stress directly disrupts organismal function. Heat stress during development can reduce immunocompetence, alter phenoloxidase activity, and impair survival in workers, queens, and drones, indicating caste-specific effects on immune defenses [23]. Heat waves can also interact with chemical stressors to suppress cellular immunity, reduce hemocyte counts, and increase oxidative stress responses, highlighting the physiological costs of combined stress exposure [24]. Thermal stress also affects honeybee behavior and colony thermoregulation. Colonies respond to sustained high temperatures by modifying fanning activity, brood distribution, and comb organization to maintain internal hive stability [25]. In addition, temperature fluctuations influence the expression of heat shock proteins, antioxidant pathways, and immune-related genes, reflecting broad molecular adjustments to thermal stress [26]. These behavioral adjustments require substantial energy investment and may alter foraging efficiency and resource allocation. Together, these findings show that heat stress influences both physiological and behavioral processes and should be considered an important component of environmental stress response models.

Water is another important but sometimes underestimated route of exposure. Honeybees collect water for thermoregulation, larval feeding, and general colony maintenance, and this behavior can bring them into contact with pesticide-contaminated guttation droplets, puddles, irrigation runoff, or standing water near agricultural fields. Ref. [27] demonstrated that honeybees may collect pesticide-laden water, especially in intensive agricultural landscapes, indicating that exposure assessment based only on nectar and pollen is incomplete. This route is especially relevant during warm periods when colony water demand increases. The hive can also retain pesticide residues. Lipophilic compounds accumulate in wax and remain available for long-term exposure. Ref. [12] reported widespread contamination of hive materials, particularly wax, by multiple pesticide residues. Ref. [28] later confirmed that wax, pollen, and bees within managed colonies often contain diverse pesticide mixtures derived from both agricultural chemicals and in-hive acaricide treatments. Therefore, exposure may continue even when bees are not actively foraging. This is especially important for brood and newly emerged adults developing inside contaminated hives. The importance of water and wax exposure varies with agricultural practices, season, and colony management. In intensive agricultural landscapes, contaminated water sources and accumulated wax residues may increase colony risk, particularly during warm periods with high water collection activity. In semi-natural landscapes, these exposure routes may be less severe because pesticide contamination is lower.

**Figure 1:**
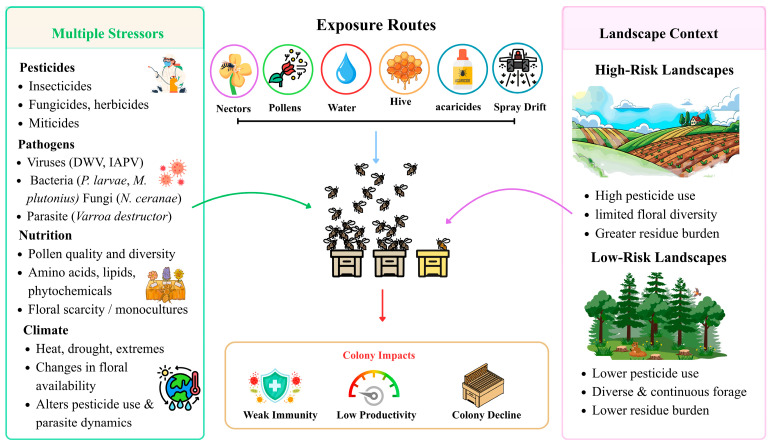
Conceptual overview of honeybee exposure pathways and colony-level impacts. Multiple environmental stressors including pesticides, pathogens, nutritional limitations, and climate variability enter the hive through nectar, pollen, water, and hive materials. These stressors interact within the colony to influence immunity, productivity, and overall colony health. The magnitude of exposure and the resulting impacts are shaped by landscape context: high-risk agricultural or urban areas increase chemical residues and reduce forage diversity, whereas low-risk semi-natural landscapes provide cleaner and more continuous resources. Arrows indicate the direction of interactions from stressors to exposure routes and subsequent colony effects.

Colonies are exposed to pesticides through the specific landscapes in which they forage, with landscape composition, diversity, and contaminant persistence directly shaping the chemical load entering the hive. Landscape composition also affects forage diversity and nutritional quality. Landscape diversity influences colony nutrition and tolerance to pathogens and pesticides. Intensive land use can reduce floral diversity while increasing pesticide exposure [29]. Therefore, landscape conditions influence both exposure level and colony resilience. Colonies in pesticide-rich and nutritionally poor environments are more vulnerable to stress. Honeybee exposure results from combined stressors, pesticides, pathogens, parasites, nutrition, and climate, entering via nectar, pollen, water, and hive materials, with landscape conditions shaping their impacts and colony responses in *Apis mellifera*. A conceptual overview of these interacting stressors, exposure routes, and landscape influences is presented in Figure 1.

## 3. Genetic Mechanisms of Detoxification

### 3.1. Detoxification Gene Families

CYP9Q, in particular CYP9Q3, is one of the most researched CYP enzymes in honeybees. This enzyme detoxifies several major pesticides, including neonicotinoids and other commonly used insecticides. CYP9Q3 function is conserved across multiple bee species, indicating an evolutionarily stable mechanism for pollinator pesticide resistance. Recombinant studies of CYP9Q3 orthologs confirmed their role in neonicotinoid detoxification across bee species [30]. The tasks and developmental stages of the bee are closely related to the expression of CYP genes. CYP gene expression varies with developmental stage and worker task. Foragers show elevated expression of CYP4G11 and CYP9Q genes, particularly in tissues exposed to environmental chemicals such as antennae and legs, suggesting roles in both detoxification and chemosensory processes during foraging [31]. Furthermore, CYP genes are significantly downregulated in larvae exposed to herbicides, which may jeopardize colony survival and development [32]. CYP336 enzymes contribute to detoxification of plant alkaloids, reflecting functional specialization within the honeybee P450 system [33]. CYP6AS enzymes represent another important detoxification pathway in honeybees. The CYP6AS subfamily metabolizes plant-derived compounds such as flavonoids, indicating a role in dietary xenobiotic processing [34]. Multiple CYP6AS genes (e.g., CYP6AS1, CYP6AS3, CYP6AS4, and CYP6AS10) are induced by honey and pollen extracts and respond to common plant chemicals, supporting their role in environmental chemical metabolism [35]. Together, the CYP9Q and CYP6AS families contribute to detoxification of both synthetic pesticides and natural phytochemicals [36]. The gut microbiota further influences CYP expression, where dysbiosis can reduce detoxification capacity and increase pesticide sensitivity [37]. Some fungicides can inhibit CYP9Q activity and act synergistically with insecticides, increasing bee mortality [38].

Recent research has demonstrated that GSTs play a direct role in the detoxification of neonicotinoid pesticides, such as imidacloprid. In particular, a defensive reaction may cause GST activity to rise at first, but prolonged or high-dose exposure inhibits it. At low imidacloprid concentrations, for example, GST activity rose to 113% after brief exposure, but at higher doses or longer exposure durations, GST activity was markedly inhibited [39]. GST expression also varies across tissues and developmental contexts, reflecting differences in exposure to environmental xenobiotics. Higher expression in gut and antennal tissues is typically observed in foragers, consistent with increased contact with external chemicals during foraging activity. These patterns, which are part of a broader regulatory response to environmental conditions, contribute to the honeybee’s ability to adjust detoxification capacity in response to chemical exposure [31]. Glutathione S transferases (GSTs) are well-characterized Phase II detoxification enzymes in insects that catalyze the conjugation of glutathione to xenobiotic substrates, aiding in their solubilization and excretion. Although some non-catalytic sequestration roles of GSTs have been reported in other insects such as *Chilo suppressalis*, such passive binding mechanisms have not been directly demonstrated in *Apis mellifera* specifically. In honeybees, GST isoenzymes have been shown to be expressed across developmental stages and induced under stress conditions, and a delta class GST (AmGSTD1) has been functionally characterized for activity toward typical GST substrates, suggesting a role in oxidative stress defense and xenobiotic metabolism; however, definitive evidence for GST-mediated sequestration of insecticides remains lacking in this species [40,41]. Therefore, while GSTs contribute to detoxification in honeybees, extrapolations regarding non-metabolic sequestration should be interpreted cautiously until direct functional validation is available.

Carboxylesterase CCE gene expression is dynamically regulated, according to recent transcriptomic analyses conducted across honeybee life stages. Strong expression of the gene has been found in detoxification tissues like the midgut, fat body, and Malpighian tubules. These tissues serve as the main locations for the clearance of xenobiotics and are comparable to the kidneys and liver of mammals [1]. Although direct functional characterization of carboxyl/cholinesterases (CCEs) in honeybees remains limited, comparative evidence from other insect taxa provides candidate mechanisms for xenobiotic metabolism that can inform hypotheses in *Apis mellifera*. For example, overexpression of specific CCE genes has been shown to enhance permethrin degradation in *Culex quinquefasciatus*, indicating that CCEs can contribute to insecticide metabolism in insects more broadly [42]. Similarly, exposure to diverse insecticides induces upregulation of multiple CCE genes in *Bactrocera dorsalis*, supporting the potential for CCE involvement in general xenobiotic responses [43]. These findings, together with reports of expressed CCE transcripts in honeybee detoxification tissues, suggest that analogous roles for CCEs in honeybees warrant targeted functional investigation rather than an assumption of conserved activity. Crucially, some honeybee CCEs have demonstrated sensitivity to particular stressors. In queens, for instance, exposure to neonicotinoids and parasites changed the midgut activity of carboxylesterase enzymes, suggesting their role in detoxification and potentially in regulating the body’s resistance to various stressors [44]. ABC transporters in honeybees are classified into several subfamilies (ABCA–ABCH) and are implicated in chemical defense and xenobiotic transport. After xenobiotics are metabolized by Phase I and II enzymes such as cytochrome P450s and glutathione-S-transferases, ABC transporters contribute to the final detoxification stage through efflux of compounds. The gut, fat body, and Malpighian tubules show high expression of ABC transporters and function analogously to vertebrate liver and kidney tissues [45]. ABC transporters are expressed in detoxification-related tissues and show developmental variation across life stages and castes, with higher expression in foragers being consistent with increased environmental exposure [46]. While comparative studies in other insects report associations between ABC transporter activity and insecticide tolerance, in honeybees this evidence remains largely correlative and based on transcriptomic responses rather than functional validation. Therefore, ABC transporters in *Apis mellifera* are currently considered candidate components of xenobiotic defense rather than confirmed detoxification mechanisms.

### 3.2. Regulation of Detoxification

The Cap‘n’collar isoform C (CncC)/Keap1 pathway is a major regulator of detoxification and oxidative stress responses in insects. In *Apis mellifera*, homologs of CncC and Keap1 have been identified, and antioxidant response element motifs are present in detoxification-related genes [22]. Studies in other insects show that activation of this pathway increases the expression of detoxification genes and enhances pesticide tolerance [47]. However, direct functional validation in honeybees remains limited.

Unlike mammals, honeybees have a notable epigenetic alteration known as DNA methylation, which primarily occurs in the mosaic pattern of gene bodies, especially exons. Particularly for genes connected to detoxification, this kind of methylation is associated with alternative splicing and active gene expression [48]. Evidence indicates that DNA methylation can affect which exons are skipped or included during mRNA processing. Enzyme function may be altered to accommodate shifting environmental toxins as a result of this direct impact on the protein isoforms generated [49]. Epigenetic cues control alternative splicing, which allows a single detoxification gene to produce multiple isoforms. These isoforms might work well with different substrates or physiological conditions. For example, splice-site usage is defined by histone modifications and DNA methylation patterns, which can result in the creation of splicing variants with changed stability or catalytic activity [50]. According to a recent study, honeybees have a transposable element-derived miRNA (ame-mir-3721-3p) that targets and suppresses the DNA methylation gene DNMT3. This regulation influences the epigenetic control of detoxification gene networks and contributes to caste-specific development, including queen differentiation, by modulating both the metabolic and detoxification pathways necessary for distinct physiological roles [51]. Another study found that the hypopharyngeal glands exhibit seasonal epigenetic regulation, with CpG methylation of growth and metabolism-related genes associated with springtime gland activation. These trends might influence the expression of detox genes and represent coping mechanisms for seasonal exposure to chemicals [52].

### 3.3. Brain Responses to Pesticide Exposure: Neurotoxicity and Behavior

While the brain is not a primary detoxification organ, it is affected by xenobiotics indirectly through oxidative stress, metabolic adaptations, and neuroimmune interactions, linking neurotoxicity with detoxification processes.

Detoxification genes are highly expressed in the midgut during all three phases of xenobiotic metabolism: Phase I enzymes, such as cytochrome P450 monooxygenases (P450s); Phase II enzymes, such as glutathione-S-transferases (GSTs) and carboxylesterases (CCEs); and Phase III transporters, such as ATP-binding cassette (ABC) proteins. The significance of the midgut throughout the life cycle is confirmed by the particular upregulation of these genes in adult bees and during larval development [1]. By increasing P450 expression in the midgut, the honeybee’s gut microbiota greatly improves its capacity for detoxification. In contrast, bees with antibiotic-induced dysbiosis show decreased P450 activity, higher pesticide accumulation, altered detoxification gene expression, and reduced survival when exposed to insecticides [37]. According to histological and transcriptome studies, exposure to pesticides like imidacloprid or atrazine damages midgut epithelial cells, reduces nutrient absorption, and modifies the expression of genes linked to detoxification. These effects weaken immune responses and increase susceptibility to infections, particularly when detoxification capacity is compromised [53]. Through the midgut, diet also affects detoxification ability. While low-quality-pollen diets make bees more susceptible to toxins, nutrient-rich pollen increases pesticide resistance by boosting detox gene expression [54]. Furthermore, the host midgut and its microbiota can work together to metabolize plant toxins like amygdalin. Microbiota-colonized bees successfully finish the detoxification process, highlighting the significance of host–microbe synergy in the midgut, while bees without a functional microbiome exhibit accumulation of intermediate toxins in the gut [55].

According to a recent transcriptome study, the fat body exhibits high expression of more than half of the known detoxification gene inventory in *Apis mellifera*, specifically genes from carboxylesterases, ABC transporters (mostly ABCC subfamily), and cytochrome P450s (particularly CYP3 and CYP4). The midgut, fat body, and Malpighian tubules exhibit the highest levels of this tissue-specific expression, indicating that these organs play a major detoxification role [1]. Furthermore, xenobiotic metabolism is directly aided by the fat body. For example, the detoxification enzyme 10-formyl tetrahydrofolate dehydrogenase (10-FTHFDH) was identified and shown to function as a dehydrogenase in honeybees. Formic acid, a common remedy for Varroa mites, is metabolized by it. In bees exposed to formic acid, this enzyme is upregulated, indicating that it plays a part in detox pathways that are probably active in the fat body [56]. Another significant molecule made by the fat body is vitellogenin, which supports resistance to oxidative stress and immunity. The upregulation of this multifunctional protein in response to microbial infections and ROS exposure highlights the dual role of the fat body in detoxification and immune defense [57]. Melittin, a cytolytic peptide best known as the main toxin in bee venom, is also produced by fat body cells. Its production increases under bacterial infection, indicating that melittin primarily functions as an antimicrobial effector within the fat body. While its activity helps neutralize pathogens, it should not be considered a direct detoxification mechanism, but rather an immune-mediated protective response [58]. When exposed to pesticides, the fat body exhibits plastic reactions. Detoxification-related genes like SOD1 and CYP9Q1-3 were markedly upregulated in the fat body of bees exposed to flupyradifurone and other agrochemicals, suggesting its function in pesticide metabolism [59]. The loss of body fat reserves caused by stress leads to an earlier transition from nursing to foraging, likely as an indirect physiological consequence of reduced energy storage. Exposure to pesticides such as spirodiclofen, which impairs fat synthesis in the fat body, further accelerates this shift [60]. A related study on stingless bees, which share physiological similarities with honeybees, demonstrated that exposure to pesticides like imidacloprid and glyphosate caused vacuolization, nuclear changes, and increased apoptosis markers in fat body trophocytes and oenocytes [61]. While informative, these findings should be interpreted cautiously for *Apis mellifera*.

Similar to the kidneys of vertebrates, honeybees’ Malpighian tubules are important excretory organs. They are necessary for the excretion of metabolic waste, xenobiotic detoxification, and the maintenance of osmotic balance. The high expression of genes associated with detoxification, including cytochrome P450s, glutathione S-transferases, and ABC transporters, indicates that these tubules directly process toxic substances that bees consume or come into contact with [1]. Detox capacity is impacted by their developmental remodeling during metamorphosis. During the early stages of pupal development, the tubules are initially non-functional and only later develop full excretory activity. This implies that young pupae’s limited detox capacity makes them more susceptible to toxins [62]. Malpighian tubules serve multiple functions in honeybees, including detoxification of xenobiotics, excretion of metabolic waste, and maintenance of osmotic balance. Histopathological alterations, such as vacuolation following sublethal exposure to pyraclostrobin and thiamethoxam, were observed in honeybees and are likely to impair both detoxification and excretory capacities under realistic exposure conditions [63]. Detox function is also compromised by infections. The Malpighian tubules’ epithelial cells are invaded and destroyed by the protozoan *Malpighamoeba mellificae*, resulting in structural deterioration that jeopardizes hemolymph homeostasis and waste excretion. Amoebiasis may result from this, decreasing the survival rate of infected bees [64,65].

Certain pesticides, including neonicotinoids and fipronil, are known to affect neural signaling in honeybees, including acetylcholine-mediated pathways in the Kenyon cells of mushroom bodies, which are critical for learning and memory. These effects vary among pesticide classes and doses and can lead to behavioral alterations even at sublethal levels [66]. According to proteomic and transcriptomic analyses, sublethal pesticide exposure induces significant neurophysiological changes in honeybee brains. Exposure to pesticides such as sulfoxaflor and carbendazim alters proteins involved in energy metabolism, neurotransmitter transport, hormonal regulation, and neurodevelopment [67,68]. Epigenetic modifications, including altered RNA methylation patterns, have also been reported following pesticide exposure, indicating sensitive molecular responses to neurotoxic stress [69]. Histological studies further demonstrate that prolonged exposure to certain pesticides can cause neuronal damage in brain regions associated with sensory integration and cognition [70]. In queens, pesticide-contaminated hive matrices are associated with differential expression of brain-related genes involved in neurodevelopment and stress response [71]. Together, these findings show that sublethal pesticide exposure can disrupt honeybee neurophysiology and behavior, with potential consequences for learning, cognition, and foraging performance. To enhance ecotoxicological evaluation instruments, in vitro models of brain tissue culture have been created. Honeybee brains can be cultured for a brief period of time under ideal media conditions to examine the effects of pesticides on Kenyon cells with little structural disorder [72].

## 4. Genetic Mechanisms of Immunity

### 4.1. Core Immune Pathways

Gram-positive bacteria and fungi are the main agents that activate the Toll pathway. This pathway is important for honeybees’ antiviral and antibacterial immunity. For instance, Toll signaling against the Israeli Acute Paralysis Virus (IAPV) depends on the peptidoglycan recognition protein PGRP-S2. This gene’s knockdown indicates that Toll is a major antiviral pathway in *Apis mellifera* because it impairs Toll activation and increases vulnerability to viral replication [73]. Additionally, the presence of gut symbionts such as *Snodgrassella alvi* modulates Toll activity, which strengthens gut immunity and increases the expression of antimicrobial peptides [74]. Gram-negative bacteria are primarily responsible for activating the immune deficiency (Imd) pathway. In the guts of honeybees, its activity has been noted, particularly in reaction to non-native microbial strains. For example, bees exhibit a more robust immune response to non-native strains of *Gilliamella*, generating reactive oxygen species (ROS) through the Duox enzyme, thereby establishing an antagonistic environment for the microorganisms [75]. Although the JNK pathway is well-characterized in insects such as *Plutella xylostella* and *Bombyx mori*, honeybee-specific evidence is limited, and its role is inferred from these related species. Based on evidence from other insects, the JNK pathway may function downstream or in coordination with Imd and Toll signaling and may participate in apoptosis, stress responses, and immune modulation; however, these roles remain incompletely validated in *Apis mellifera*. Along with Toll and Imd, the JNK pathway is upregulated during bacterial and fungal infections in insects such as *Plutella xylostella* and *Bombyx mori* [76]. The Jak/STAT pathway is implicated in viral defense and systemic immune responses, and in *Apis mellifera*, certain components show altered expression upon microbial exposure; however, detailed functional characterization in honeybees remains limited. In insects, interactions between gut bacteria and host cells can activate the JAK/STAT signaling pathway, which may contribute to the regulation of systemic immune responses and longer-term immune priming [77]. Together, these pathways are proposed to contribute to a multi-layered immune network in honeybees, although some pathway interactions remain inferred from comparative insect studies. Although bees have relatively few immune genes, their activation is highly regulated and vulnerable to environmental stressors, viral infections, and microbial interactions, highlighting the evolutionary significance of these genes [78].

Peptides, which are primarily produced by adult workers, disrupt microbial membranes and prevent pathogens from carrying out vital biological tasks, acting as humoral effectors of immunity [79]. Intracellular immune signaling pathways, particularly the Toll and Imd pathways, tightly control the expression of AMP genes. The Imd pathway controls the synthesis of abaecin and hymenoptaecin, whereas the Toll pathway primarily activates the genes encoding defensins and apidaecins. When pattern recognition receptors (PRRs) identify pathogen-associated molecular patterns (PAMPs), like bacterial peptidoglycans, these pathways are activated [80]. Developmental stage, behavioral caste (forager vs. nurse), exposure to environmental stressors like pesticides, and infection by viruses like deformed wing virus (DWV) or pathogens like *Nosema* spp. are some of the factors that dynamically affect AMP synthesis rather than keeping it constant. For instance, foragers exhibit significantly greater levels of AMP gene expression than nurse bees, particularly in tissues involved in nectar processing such as the mandibular and hypopharyngeal glands [81]. One important modulator of AMP expression in the gut microbiota is lactic acid bacteria (LAB). These symbionts can strengthen immune responses and increase resistance to dangerous infections by activating AMP genes. This mutualistic relationship demonstrates how endogenous peptides and microbial partners work together to preserve honeybee health [2].

#### 4.1.1. Natural Antiviral RNAi Responses

In honeybees (*Apis mellifera*), RNA interference (RNAi), a sequence-specific gene-silencing pathway initiated by double-stranded RNA (dsRNA), is an essential antiviral defense mechanism. Among the main causes of colony losses, this system aids bees in fending off infections from a variety of RNA viruses, including Sacbrood Virus (SBV), Israeli Acute Paralysis Virus (IAPV), and deformed wing virus (DWV). Dicer-2 breaks down viral dsRNA into small interfering RNAs (siRNAs), and Argonaute-2 (Ago2) uses these siRNAs to direct the breakdown of complementary viral RNAs, making up the fundamental machinery of RNA interference. This process can significantly reduce viral replication and prevent obvious symptoms of infection [82].

#### 4.1.2. Experimentally Applied dsRNA Interventions

Oral administration of virus-specific dsRNA has been shown in experiments to effectively lower viral loads and increase infected bee survival. For example, dsRNA targeting acute bee paralysis virus (ABPV) genes dramatically reduced bee mortality and virus replication [83]. Similar results have been noted for Sacbrood Virus, wherein ingestion of dsRNA decreased larval mortality in *Apis cerana* [84]. RNA interference (RNAi) in honeybees can be transmitted socially and systemically, with dsRNA consumed by nurse bees and passed to larvae through worker and royal jelly, resulting in gene knockdown in the next generation. While this transmissible RNA pathway suggests a potential form of colony-level social immunity, further experiments are required to confirm its protective effects at the colony scale [85]. The viral load, the delivery method (injection vs. feeding), the bee’s developmental stage, and the target tissue can all affect how effective RNA interference is. Downregulation of RNAi-related genes is observed in bees infected with high virus loads, indicating that certain viruses may inhibit this immune pathway in order to evade detection [86]. Furthermore, honeybees have antiviral genes specific to viruses, such as bee antiviral protein-1 (bap1), which co-expresses with Argonaute-2 and works in tandem with RNA interference to regulate viral infections. This gene reflects continuous adaptation to viral pressures and seems to be evolutionarily exclusive to hymenopterans [87].

### 4.2. Organ-Specific Immune Functions

The gut epithelium not only acts as a barrier against pathogens but also coordinates with detoxification pathways to modulate systemic immune responses. Damage or dysbiosis in this tissue can therefore simultaneously compromise chemical tolerance and immunity, highlighting its central role in maintaining colony health.

The midgut epithelium is the main site of infection for honeybees infected by pathogens such as *Nosema ceranae*. Intestinal stem cells (ISCs), which are vital for gut renewal and homeostasis, are inhibited from proliferating and enterocytes are damaged by the infection. This damage impairs the bee’s ability to repair epithelial tissue, reducing its longevity and stress tolerance significantly [88]. Additionally, gut microbes actively influence immune responses in the epithelium. The gut symbiont *Frischella perrara* was shown to trigger a localized immune reaction called the melanization response, forming “scab-like” structures at the pylorus. These responses involve upregulation of the melanization cascade, antimicrobial peptide genes, and pattern recognition receptors in the gut epithelium [89,90]. It was demonstrated that *Lactobacillus apis*, another advantageous symbiont, activates the Toll pathway and increases the synthesis of antimicrobial peptides like apidaecin, which aid the bee in fending off infections from pathogens like *Hafnia alvei*. Certain gut bacteria have distinct surface proteins (like S-layer proteins) that directly affect immune gene expression, according to this strain-specific immune modulation [91]. Finally, dysbiosis or damage to the gut epithelium whether due to pathogens like *Serratia marcescens* or toxic substances can lead to increased permeability and mortality. Honeybee studies using gut dysbiosis models show that chemical-induced gut damage suppresses critical detox and immune pathways while disrupting the microbiota. Interventions such as anti-inflammatory drugs or beneficial bacteria, however, can restore immune stability and gut barrier function [92].

Crucially, when a microbial infection occurs, the expression of the Vg gene is markedly increased in the fat body, indicating a direct function in immune defense [57]. The fat body works in tandem with hemocytes, or immune blood cells, in addition to AMPs, to regulate systemic immune responses. The fat body specializes in the synthesis and secretion of humoral effectors such as AMPs, whereas hemocytes are mainly involved in phagocytosis [93].

Prohemocytes, granulocytes, and plasmatocytes are among the different hemocyte types found in honeybees, with granulocytes and plasmatocytes exhibiting the highest immunological activity [94]. These cells maintain internal homeostasis by phagocytosing bacteria and apoptotic cells. Hemocyte numbers and activity increase in response to microbial challenges, such as exposure to *Escherichia coli* lipopolysaccharides (LPSs), but are highly sensitive to chemical exposure; for instance, imidacloprid reduces hemocyte function, particularly in bees with compromised immunity [95]. Observed differences among nurses, foragers, and queens in hemocyte count and phagocytic activity reflect a combination of age, caste-specific tasks, nutritional state, and prior exposure to stressors [96,97]. The hemolymph contains soluble immune factors like phenoloxidases (POs) that drive melanization, a key defense against pathogens. Sublethal exposure to pesticides such as thiacloprid and clothianidin impairs this process, reducing both hemocyte viability and overall immunocompetence [98]. Finally, the development of reference molecular markers facilitates hemocyte identification and enables standardized studies of immune function in ecological and toxicological contexts [99].

A growing body of research suggests the potential existence of a gut–brain–immune axis in honeybees, although direct evidence remains limited. For example, Zhang et al. (2022) observed that gut bacteria, such as *Lactobacillus* and *Gilliamella*, can influence circulating metabolites and neurotransmitter levels in honeybee brains, which may impact behavior and immunological signaling [100]. These findings imply a possible metabolic and immunological bridge between the gut and neural systems, but further validation is needed. Related work in bumblebees indicates that chronic activation of humoral immune responses can reduce foraging efficiency and cognitive flexibility [101]. While suggestive, these effects have not been directly demonstrated in *Apis mellifera*. Additionally, the vitellogenin–hypopharyngeal gland axis in honeybees appears to link immune function and brain-related processes, as ingestion of heat-killed pathogens increases defensin-1 and vitellogenin levels, proteins implicated in immune priming and potentially in neural function [102]. Collectively, these studies support the hypothesis that the immune and neurological systems in honeybees may interact through molecular cues, but definitive mechanistic evidence for a functional gut–brain–immune axis in *Apis mellifera* remains to be established.

### 4.3. Pathogen-Specific Responses

Deformed wing virus (DWV) and Israeli Acute Paralysis Virus (IAPV) are major viral threats to honeybee colonies because they connect individual infection with colony-level decline. DWV is strongly amplified by *Varroa destructor*, which functions not only as a vector but also as a driver of viral replication and immune disruption. High DWV loads are associated with wing deformities, shortened lifespan, impaired cognition, and reduced colony performance, whereas colonies with low mite population growth often show lower viral loads, suggesting that mite resistance and viral resistance are mechanistically linked [103,104]. IAPV produces acute paralysis and rapid worker mortality, but its impact is best understood within a broader viral-stress framework rather than as an isolated infection. Co-detection of IAPV with DWV suggests that mixed viral infections may intensify morbidity by increasing the immune burden on infected bees. Age- and caste-related differences in IAPV levels further indicate that behavioral role, exposure history, and physiological condition shape viral susceptibility within the colony [105]. At the molecular level, viral infections interfere with host defense through immune and epigenetic pathways. DWV can suppress apoptosis-related genes, allowing infected host cells to survive long enough to support viral persistence, while IAPV infection is associated with early DNA methylation changes that may reflect epigenetic immune regulation [106]. These responses show that viral pathogenesis is not limited to direct tissue damage; it also involves manipulation of host immune signaling, cell survival, and gene regulation. The gut also represents an important interface for viral invasion and immune protection. Gut-binding peptides that block DWV and IAPV attachment to epithelial receptors can reduce viral load and limit systemic infection, indicating that epithelial barriers are central to antiviral defense [107]. Beyond the colony, DWV and IAPV can infect non-Apis arthropods, including yellow-legged hornets and other insects, creating opportunities for cross-species spillover [108,109]. Such spillover may increase environmental viral reservoirs and strengthen transmission pressure on managed and wild pollinators, especially when colonies are already weakened by mites, poor nutrition, or chemical exposure [110].

#### 4.3.1. American Foulbrood (AFB)

American foulbrood (AFB) is caused by the spore-forming, Gram-positive bacterium *Paenibacillus larvae*. It is highly contagious due to its persistent spores, which can persist for decades. After being consumed, the spores germinate in the midgut of the larva, penetrate the epithelial barrier, and cause a systemic infection that, once the cell is sealed, results in death. AFB is usually lethal and, if left unchecked, can destroy the colony. Honeybee larvae respond by triggering humoral and cellular immunity, which includes hemocytes producing more nodules and the expression of antimicrobial peptides such as apolipophorin III. Phospholipase A2 enzymes, which are essential for establishing efficient defenses, control these reactions [7].

#### 4.3.2. European Foulbrood (EFB)

*Melissococcus plutonius*, which colonizes the larval gut without piercing the epithelium, is the cause of European foulbrood (EFB). Larvae pass away earlier in development, usually before cell capping. Other bacteria that act as secondary invaders, such as *Enterococcus faecalis* or *Achromobacter eurydice*, frequently co-infect EFB [111]. Both AFB and EFB activate overlapping immune pathways, according to comparative immune studies; however, *P. larvae* typically elicit stronger systemic responses, such as prophenoloxidase activity and broader antimicrobial responses [112]. It is interesting to note that infected prepupae show reduced protease activity, which could impede a healthy immune response and increase bacterial virulence [113].

#### 4.3.3. Microbiota and Experimental Interventions

Recent research highlights that microbiota-targeted interventions may enhance honeybee resistance to bacterial pathogens by strengthening immune regulation, limiting pathogen colonization, and improving physiological resilience. Beneficial gut-associated bacteria, particularly *Lactobacillus* and *Bifidobacterium* species, can stimulate immune activity, support antimicrobial responses, and reduce mortality associated with American foulbrood (AFB) and European foulbrood (EFB), suggesting that microbiome stabilization represents a promising strategy for colony health management [114]. In contrast, bacteriophage therapy remains largely at the experimental stage, showing promise in laboratory studies for selectively reducing bacterial loads but requiring further validation under field conditions [115]. Similarly, nano-silver compounds have been tested experimentally to lower bacterial burden and improve larval survival rates [116], but concerns regarding dosage, toxicity, and environmental safety mean these are not yet ready for routine field use.

An infection with *N. ceranae* causes chronic immunosuppression and energy stress in adult bees by suppressing important immune pathways such as Toll and Imd and downregulating genes that encode antimicrobial peptides. Because of their weakened physiological resilience and early onset of foraging activity, infected bees exhibit increased lipid depletion, elevated metabolic demand, and a shorter lifespan [117,118]. Recent molecular research has demonstrated that long non-coding RNAs (lncRNAs) and circular RNAs (circRNAs) in infected midguts control phagosome activity, endocytosis, and immune signaling, exposing intricate host responses that go beyond classical immunity [119]. In controlled laboratory experiments, delivery of RNAi molecules targeting the parasite’s antioxidant systems via engineered gut symbionts such as *Snodgrassella alvi* has been shown to reduce *N. ceranae* spore loads by up to 99.8% [120]. While promising as a symbiont-based biocontrol strategy, this approach requires further evaluation under field conditions to confirm efficacy, safety, and practicality.

#### 4.3.4. Gut Microbiota and Pathogen Interactions

Silencing the bee’s naked cuticle (nkd) gene, which is a negative regulator of immune signaling, is another host-targeted tactic. This method dramatically lowers spore replication in infected bees and increases the production of antimicrobial peptides [121]. Long-term gut health impairment results from *N. ceranae*’s disruption of digestive homeostasis, which also damages gut stem cells and diminishes epithelial renewal capacity [88]. Early *N. apis* infections have physiological effects as well as changing foraging behavior, causing bees to fly more frequently and for shorter periods of time, potentially compromising pollination and productivity [122].

In bees, mites inhibit both humoral and cellular immunity. They accomplish this by disrupting NF-κB signaling, an essential immune pathway in insects. This suppression promotes DWV replication, which raises viral loads and increases mite reproduction, which is a mutualistic symbiosis between the virus and mite that deteriorates bee health [123]. Increased DWV replication has been observed in experiments where the bee hemolymph is removed, indicating that feeding itself may be a contributing factor to immune destabilization [124]. Certain populations of honeybees, such as *Apis mellifera scutellata* and *Apis cerana*, exhibit inherent resistance to *Varroa destructor*. While both species employ immune gene upregulation, increased grooming, and hygienic behaviors, the specific efficacy and expression of these mechanisms can vary between species. For example, *Apis cerana* shows more robust grooming efficiency and reduced mite reproduction compared to *Apis mellifera scutellata*, reflecting species-specific adaptations [125,126]. This distinction emphasizes that while overarching resistance strategies are similar, their relative contributions and effectiveness may differ across honeybee species. Multiple lines of resistance have been found in bees with elevated levels of antimicrobial peptides (AMPs) such as hymenoptaecin and defensin-2, as well as increased hemocyte concentration, thanks to selective breeding programs that target low-Varroa-growth traits [127]. Gene expression studies in *Apis cerana* have revealed metabolic adaptations and immune effectors that contribute to resistance [128]. The importance of integrated pest management (IPM) techniques is highlighted by the pervasive infestation of *Varroa destructor* and increasing acaricide resistance. Candidate approaches include biological control agents, RNAi-based mite control, and natural product treatments such as *Jatropha oil*. While *Jatropha oil* has shown promise in reducing mite loads without harming bee brood [129], its efficacy, safety, standardization, and field validation remain limited. Similar caution is warranted for other initially promising treatments. For example, lithium chloride showed strong systemic acaricidal activity against *V. destructor* in early experimental studies, suggesting potential as an anti-Varroa treatment [130]. However, concerns regarding dosage, delivery, honeybee safety, brood effects, and possible colony-level side effects have limited its practical application and highlight the need for careful toxicological and field evaluation before implementation. Further control trials are therefore needed to ensure consistent performance and to evaluate potential ecological and colony-level risks under diverse environmental conditions.

## 5. Integration of Detoxification and Immunity

In honeybees, detoxification and immunity are closely related systems that share many physiological resources and are triggered by similar stress signals. Oxidative stress is one of the most significant connections between them. Reactive oxygen species are produced more frequently when exposed to pesticides, infections, and other environmental stressors. These molecules serve as signals that initiate protective reactions as well as harmful effects of stress. Doublet et al., 2015 [131], state that the gut is a crucial organ for both chemical tolerance and disease resistance since it is a significant location where diet, microbiota, and infection interact. Raymann et al., 2017 [132], state that disruption of the gut microbiota increases mortality in bees exposed to opportunistic infection. Further research has shown that microbial disruption can also change susceptibility to pesticides, establishing a connection between immune stability and detoxification through the gut environment. Impairment at this site can concurrently decrease food intake, weaken local immune responses, and change systemic stress physiology since the gut epithelium is directly exposed to polluted nectar and pollen.

Oxidative stress in honeybees acts primarily as a mediator linking detoxification and immune responses. Exposure to pesticides and other xenobiotics elevates reactive oxygen species (ROS), which in turn activate antioxidant defenses, including superoxide dismutase, catalase, and glutathione-dependent pathways, as well as vitellogenin-associated protection, to maintain cellular homeostasis [133]. This ROS-mediated signaling modulates immune pathways, such as antimicrobial peptide production, and coordinates metabolic allocation to balance detoxification and immunity [134]. Furthermore, age-related changes in antioxidant capacity influence both the susceptibility to oxidative damage and the efficiency of detoxification and immune responses, highlighting ROS as central integrators of stress effects rather than mere consequences or isolated causes [135]. Recent studies by Christen et al., 2016 [136], showed that long-term exposure to thiamethoxam modifies gene expression patterns linked to metabolism and stress reactions in the honeybee brain, suggesting that broader physiological changes accompany detoxifying costs. The hypothesis that chemical stress is not limited to detoxifying enzymes alone is supported by [137], who showed that pesticide exposure modifies metabolic and immune-related gene expression in worker bees. By demonstrating that clothianidin can weaken antiviral defenses by adversely influencing the NF-κB immune signaling pathway, hence enabling viral multiplication, ref. [138] made a significant contribution. This work unequivocally showed that, in addition to generating overall toxicity, pesticide stress can decrease immune function at the molecular level. Additionally, ref. [98] showed that pesticide mixtures can alter honeybee survival and immune responses in ways that differ from single-compound exposure, highlighting the fact that immunological disruption and detoxifying requirements frequently happen at the same time.

Honeybee immunocompetence is strongly influenced by energetic and metabolic constraints. Nutritional limitation reduces the energy available for immune defense, while pathogen challenges such as Nosema infections further increase metabolic demand, impairing physiological performance [13,139,140]. Age- and task-related changes modulate metabolic investment and antioxidant capacity, making foragers more susceptible to stress [141]. Exposure to neonicotinoids, including imidacloprid, thiacloprid, and fipronil, compromises immune signaling pathways such as NF-κB, which elevates susceptibility to Nosema infection and increases mortality [142,143]. Chronic consumption of pesticide-contaminated food exacerbates Nosema intensity, highlighting the cumulative effects of combined chemical and biological stressors on honeybee immunity [144].

Studies by Amdam et al., 2005 [145], and Corona et al., 2007 [146], showed that vitellogenin produced in the fat body plays a role in oxidative stress resistance, longevity-related processes, and physiology connected to reproduction. Because of this, the fat body plays a crucial role in striking a balance between immunological protection and the expense of detoxifying. The fat body takes part in xenobiotic metabolism when bees are exposed to pesticides, but it also needs to promote AMP synthesis and metabolic reorganization when an illness develops. A mechanistic foundation for trade-offs is created by such simultaneous needs, particularly in situations involving repeated chemical challenges or nutritional stress. In a study by Gätschenberger et al., 2013 [147], significant humoral components in the hemolymph, specifically antimicrobial peptides and phenoloxidase-associated activities, are involved in immunological activation in honeybees. Therefore, rather than being distinct processes, changes in hemolymph composition reflect the combination of immunity and detoxification. At this systemic level, pesticide-induced oxidative stress, altered metabolite profiles, and decreased immunological effector activity can all be seen. This kind of cross-tissue coordination is crucial for comprehending why sublethal stresses can have disproportionate biological effects, according to [148]. As a result, the ability of many organs to effectively coordinate oxidative balance, metabolic allocation, and defense signaling under stress is just as important to honeybee resilience as the efficacy of particular detoxification genes or immunological pathways. Activation of both immune defenses and detoxification pathways imposes substantial energetic costs on insects, leading to metabolic trade-offs when resources are limited. Immune activation requires the rapid synthesis of immune effectors, increased glycolysis, and the mobilization of energy reserves, which redirects nutrients such as glucose and amino acids toward immune cells at the expense of other physiological functions—including growth, storage, and detoxification processes [149,150]. In insects, the fat body serves as a central hub for both energy storage and immune function, and during infection there is a systemic metabolic switch that prioritizes immune signaling and antimicrobial peptide production over anabolic processes, thereby reallocating energy from carbohydrate and lipid storage toward defense mechanisms [150]. Similarly, the energetically demanding activity of detoxification enzymes, such as cytochrome P450s, glutathione-S-transferases, and associated antioxidant systems, increases overall metabolic rate and depletes energy reserves, which can limit the resources available for mounting a robust immune response, particularly under chronic xenobiotic exposure [151]. Collectively, these findings suggest that simultaneous immune activation and detoxification may impose competing energetic demands in honeybees, consistent with broader patterns reported in other insects.

In honeybees and other insects, cellular maintenance mechanisms such as heat shock proteins (HSPs), proteostasis networks, and antioxidant defenses act as a fundamental support layer that preserves homeostasis and sustains the functional integrity of both detoxification and immune pathways under diverse environmental stressors. HSPs, including members of the HSP70, HSP90, and small HSP families, are molecular chaperones that stabilize protein structure, assist in folding, prevent aggregation, and maintain protein functionality when cells are challenged by thermal extremes, xenobiotics, pathogens, or oxidative stress [18,22]. This proteostasis network which integrates the heat shock response with unfolded protein responses and oxidative stress responses ensures that critical enzymes and signaling components of detoxification (e.g., cytochrome P450s and GSTs) and immune effectors (e.g., antimicrobial peptides and signaling kinases) remain functional under stress, thereby buffering physiological systems against collapse [22]. In addition to chaperoning proteins directly, HSPs interact with antioxidant systems to mitigate reactive oxygen species, which are generated during pesticide detoxification and immune activation, linking cellular maintenance with broader organismal resilience [21]. Evidence from proteostatic stress studies in honeybee tissues further demonstrates that sHSPs are upregulated by multiple stress pathways and could serve as core stress biomarkers, highlighting their central role in maintaining cellular homeostasis across stress contexts [22]. Integrating these cellular maintenance processes as a transversal layer within the honeybee stress response framework therefore provides a more comprehensive representation of how detoxification and immunity are supported at the molecular level, reinforcing organismal resilience to multifactorial environmental challenges.

## 6. Multi-Stressor Interactions

Instead of separate exposures, the interplay of various stressors has a significant impact on honeybee health. This interaction is particularly noticeable when pesticides and diseases coexist. Ref. [13] demonstrated how immunological competency is influenced by dietary status, offering an early warning that the effects of infection cannot be understood in isolation from other environmental stressors. Studies have shown that sublethal exposure to pesticides such as fipronil, thiacloprid, and clothianidin can compromise antiviral and antifungal defenses, including NF-κB signaling, increasing susceptibility to *Nosema ceranae* and deformed wing virus [138,144,145,146,147,148,149]. These findings highlight that combined chemical and pathogen stressors impose higher physiological costs than individual stressors, underscoring the importance of evaluating multi-stressor effects within integrated exposure and colony context frameworks.

Sublethal exposure to neonicotinoids and other pesticides interacts with pathogen infections to elevate mortality and impair honeybee health beyond the effects of individual stressors. Field evidence shows that parasites, pesticides, and additional environmental stressors co-occur, creating cumulative physiological loads that are often underestimated in laboratory single-factor studies [9,131].

Nutrition plays a critical modulatory role, as pollen quality and diversity directly influence immunocompetence, detoxification capacity, and overall physiological resilience [14]. In nutritionally limited environments, bees may lack sufficient protein, fats, minerals, and phytochemicals to support effective immune and detoxification responses, exacerbating the negative impacts of chemical and biological stressors. Colonies located in intensively farmed landscapes often experience reduced pollen and nectar diversity, leading to poorer nutritional status, which can compromise immune function and increase vulnerability to pesticides and pathogens [14,152,153]. Pollen availability alters physiological reactions to stress, as ref. [154] showed, supporting the notion that toxins cannot be assessed without dietary context. Additionally, ref. [155] demonstrated how pesticide exposure and nutritional stress can interact to reduce honeybee performance, supporting the idea that resource availability plays a role in the biological consequences of toxic exposure. In the end, these interactions have an impact on both individual and colony survival. Henry et al., 2012 [156], demonstrated that thiamethoxam exposure reduced the likelihood that exposed workers would return to the colony by impairing foragers’ capacity to find their way home. Working on bumblebees but having a significant impact on the broader framework of pollinator stress, Whitehorn et al., 2012 [157], demonstrated that long-term pesticide exposure can impede colony growth and reproductive performance, which helped shape subsequent honeybee studies on sublethal impacts. Ref. [158] discovered that long-term exposure to neonicotinoids in the field decreased queen production and colony performance in honeybees in particular. These findings demonstrate that stress impacts can manifest as behavioral disruption, decreased brood care, decreased food input, diminished colony growth, or poor reproduction rather than as rapid worker mortality. Multiple stressors, including pesticides, pathogens, and nutritional limitations, interact over time to compound colony-level effects, reducing resilience, impairing immunity, and affecting survival and reproduction.

## 7. Biomarkers and Predictive Indicators

Transcriptomic, enzymatic, proteomic, and microbiome-based biomarkers provide complementary approaches for detecting sublethal stress in honeybees before visible colony decline occurs. Rather than reflecting isolated physiological events, these biomarkers collectively indicate how detoxification, immunity, metabolism, and neural regulation respond to environmental stressors. Transcriptomic biomarkers are particularly sensitive indicators of early physiological disruption because environmental stress rapidly alters the expression of genes associated with detoxification, oxidative balance, neural signaling, and immune regulation. Pesticide exposure modifies transcriptional profiles linked to stress response, metabolism, and neurophysiology, while pathogen infection alters immune-related pathways including antimicrobial peptides and antiviral defense genes [136,137,138,159,160]. These transcriptional changes are mechanistically important because they reveal how colonies allocate physiological resources under combined chemical and biological stress.

Enzymatic biomarkers provide a functional measure of detoxification efficiency and oxidative stress status. Enzymes such as glutathione S-transferase, catalase, superoxide dismutase, and phenoloxidase reflect the capacity of bees to maintain redox balance, neutralize xenobiotics, and sustain immune activity under stress conditions [161,162]. Altered activity of these enzymes frequently precedes visible pathology, making them useful indicators of physiological instability during chronic environmental exposure.

Proteomic and kinomic biomarkers further strengthen predictive assessment by linking molecular signaling pathways with colony resilience traits. Vitellogenin is especially important because it integrates oxidative stress resistance, immunity, longevity, and metabolic condition, thereby functioning as a broad indicator of colony health [134,141]. Kinomic and proteomic analyses additionally reveal signaling signatures associated with disease resistance, social immunity, and Varroa tolerance, supporting their potential use in selective breeding and resilience assessment [145,163].

Microbiome composition has also emerged as a powerful indicator of colony condition because gut microbial communities directly influence nutrition, detoxification, immune regulation, and pathogen resistance. Disturbance of core bacterial taxa such as *Gilliamella*, *Snodgrassella*, *Lactobacillus*, and *Bifidobacterium* is associated with increased susceptibility to pathogens and environmental stress [132,164].

**Figure 2:**
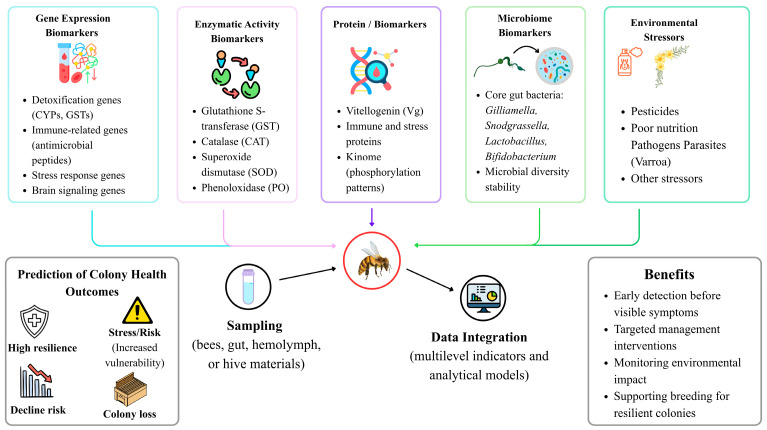
Integrated biomarker framework for honeybee health assessment.

More recently, ref. [165] demonstrated that quantitative microbiome profiles can predict winter colony loss, offering compelling evidence that microbial markers can be used to anticipate colony destiny in addition to describing stress after it occurs. The transition from descriptive microbiology to predictive colony diagnosis is significant. Instead of depending on a single metric, it is necessary to integrate these many classes of biomarkers in order to anticipate colony health and collapse. The intricate in-hive pesticide exposome was described by [28], demonstrating that colonies are exposed to many residues that can concurrently affect immune, microbiome, and detoxification pathways. In these circumstances, distinct aspects of colony stress are captured by gene-expression markers, enzyme activity, protein signatures, and microbiome composition. Additionally, ref. [140] showed that colonies in agriculturally intensive and nutritionally deficient environments exhibit quantifiable physiological deterioration, indicating that prediction techniques need to take into consideration both internal molecular cues and exterior ecological context. Therefore, indications that show both exposure and the colony’s biological ability to correct for that exposure are the most instructive. Research collectively demonstrates that indices of honeybee health include microbiome structure, kinome activity, proteins, enzymes, and gene expression. When connected to colony characteristics, these indicators can forecast decline before permanent damage happens by identifying early stress impacts on immunity, detoxification, and physiology. The relationships between molecular biomarkers, environmental stressors, and colony-level outcomes are summarized in Figure 2.

Honeybee responses to environmental stress can be detected early using multiple biomarker classes. Gene expression markers indicate changes in detoxification, immunity, and neural pathways, while enzymatic biomarkers reflect oxidative stress and detoxification capacity. Protein and kinome indicators, such as vitellogenin, reveal physiological resilience, and microbiome composition reflects colony health and metabolic balance. Together, these integrated biomarkers enable early detection of sublethal stress and prediction of colony performance and collapse risk in *Apis mellifera*.

## 8. CRISPR and Genomic Strategies for Honeybee Resilience

### 8.1. Editing Tools and Functional Validation

CRISPR/Cas9 provides a useful tool for functional validation of candidate genes in *Apis mellifera*, but its relevance to honeybee resilience should be framed cautiously. In honeybees, CRISPR/Cas9 has been used to generate heritable mutations, including disruption of the *Amyellow-y* gene, demonstrating the feasibility of genome editing in this species. Embryo-based Cas9 and guide RNA delivery methods have also produced efficient biallelic mutations, providing a practical foundation for testing candidate genes involved in development, stress response, detoxification, or immunity [166]. However, direct CRISPR-based validation of honeybee detoxification and immune genes remains limited. Therefore, future applications should focus on experimentally testing candidate genes identified from transcriptomic, proteomic, microbiome, and toxicological studies under biologically relevant stress conditions. Beyond honeybees, CRISPR/Cas9 genome editing has been widely used in other insects, such as *Drosophila melanogaster* and the jewel wasp *Nasonia vitripennis*, to investigate gene roles in detoxification, immune signaling, and host–pathogen interactions, highlighting transferable approaches and experimental designs that can inform functional studies in honeybees [167,168]. Systematic reviews further underscore the utility of CRISPR in targeting insect detoxification gene repertoires, enabling precise manipulation of xenobiotic resistance mechanisms that can be applied to pollinator species with appropriate adaptation of protocols [169]. Collectively, these gene editing advances provide a framework for future CRISPR-based functional validation of candidate detoxification and immune genes in *Apis mellifera*, while emphasizing the need for optimized delivery, screening, and phenotypic assessment strategies tailored to honeybee biology.

### 8.2. Genetic Markers and Selective Breeding for Resilience

Single-nucleotide polymorphisms (SNPs) have been linked to traits such as immune response, chalkbrood tolerance, and Varroa resistance in recent genomic work [170]. Among these, hygienic behavior is a well-established trait already employed in practical honeybee breeding programs, in which workers remove sick or parasitized brood to reduce disease spread; this trait is heritable and can be enhanced by focused selection [171]. Grooming behavior, which helps protect against *Varroa destructor*, is another trait under active selection [172]. In contrast, phosphorylation signatures identified in honeybee pupae that predict Varroa tolerance represent emerging molecular markers from kinome analyses [173] and remain largely experimental, offering the potential to accelerate breeding programs once validated. Overall, current breeding practices integrate behavioral traits such as hygienic and grooming behaviors, while molecular markers are being developed to refine selection strategies for enhanced colony resilience. In a similar vein, alterations to the gut microbiota appear promising: colonization with advantageous bacteria such as *Bartonella apis* and *Apilactobacillus kunkeei* stimulated immune gene expression and detoxification enzyme activity, enabling bees to tolerate exposure to toxic nectar [5]. Exposure to pesticides like imidacloprid or ethiprole causes immune genes and detoxification enzymes (like glutathione S transferase and cytochrome P450s) to be upregulated at the molecular level. This reveals possible genetic pathways that can be targeted in breeding or genetic engineering for resilience [174,175]. Additionally, transcriptome studies demonstrate how agrochemicals like benomyl alter immune and detoxification pathways, indicating potential targets for enhancing stress resilience [176].

## 9. Future Directions and Research Gaps

The need to make a clear distinction between mechanisms inferred from other insects and those directly shown in *Apis mellifera* is a major research goal in honeybee biology.

Honeybee (*Apis mellifera*) resilience to climate change requires concerted ecological investigation into the effects of rising temperatures on colony survival, pollination networks, and adaptive potential across subspecies and landscapes. Climate-driven shifts in habitat suitability are predicted to alter the geographical distribution of honeybee apiaries, with bioclimatic variables such as temperature seasonality and radiation strongly influencing future suitability for honeybee populations, underscoring the need to identify and conserve genotypes adapted to specific climatic conditions [177,178]. Chronic heat stress has been shown to disrupt foraging motivation and related physiological functions critical for colony growth, suggesting that sustained temperature increases may compromise food collection and ecosystem services [24]. Climate change also threatens the synchrony of plant–pollinator interactions, with weather variability and phenological shifts potentially decoupling honeybee foraging activity from floral resource availability, thereby destabilizing pollination networks [179]. Moreover, warming winters and warmer seasonal conditions negatively affect overwintering survival and pollination service continuity, highlighting the importance of long-term monitoring across seasons and climates to inform adaptive management and mitigation strategies [178,180]. Integrating ecological, physiological, and distributional studies will be essential for developing targeted breeding and conservation strategies that enhance honeybee resilience to ongoing climatic pressures.

Many suggested regulatory mechanisms still require direct functional validation in honeybees, despite the fact that comparative studies have been useful in identifying potential pathways involved in detoxification, immunity, stress signaling, and host–microbe interaction. Ref. [181] demonstrated how the immunological repertoire of honeybees differs significantly from that of other insects, highlighting the need for caution when extrapolating from model species. Similar observations have been made in more recent assessments, such as [182], finding that while gene expression investigations have found numerous potential responses to infections and pesticides, transcript quantity by itself does not prove causal function. This is particularly crucial for signaling intermediates, detoxifying regulators, and epigenetic pathways that are often described based on correlated expression patterns or homology. Therefore, additional honeybee-specific validation employing RNA interference, gene knockdown, tissue-specific tests, biochemical characterization, and, when practical, gene-editing techniques that directly evaluate potential activities under realistic stress circumstances is required for future work.

The integration of molecular datasets across biological scales is another significant gap. Few studies have integrated proteomic, metabolomic, epigenomic, and microbiome data from the same biological context with transcriptome changes in response to infections, pesticides, diet, or microbiome perturbation. Simone-Finstrom et al., 2016 [183], showed that no single molecular layer can adequately describe the interconnections between genes, physiology, behavior, and environment that result in colony health. The increasing need for system-level methods that may connect host gene regulation with microbial community dynamics, metabolite profiles, and colony performance characteristics was further emphasized by [184]. Because transcriptional responses do not always directly correlate to enzyme activity, protein abundance, microbial stability, or long-term colony results, multi-omics integration is particularly crucial. In order to identify causal biomarkers, regulatory nodes, and cross-system interactions that control resilience more precisely, future research will benefit from experimental designs that evaluate many omics layers simultaneously. The discrepancy between laboratory results and actual colony conditions is a recurring problem in the field. Because they enable control over exposure dose, timing, and co-stressor presence, laboratory trials are crucial for mechanistic resolution, yet they frequently oversimplify the ecological complexity that influences honeybee reactions in natural settings. Sublethal pesticide exposure can change foraging behavior under field-relevant settings, as demonstrated by [156], showing that effects observed in individual bees can scale into ecologically significant outcomes. However, refs. [185,186] noted that when laboratory toxicity studies fail to take into consideration variations in nutrition, weather, social buffering, floral diversity, pathogen load, or repeated low-dose exposure, they may either overestimate or underestimate field risk. This discrepancy is nonetheless significant because social organization, division of labor, and compensating behavior, all of which are challenging to replicate in lab tests, are critical components of colony resilience. Therefore, future studies must use semi-field designs, realistic pesticide mixes, changing diets, and realistically occurring pathogen pressures to strengthen the link between mechanistic laboratory data and field-based colony observations.

**Figure 3:**
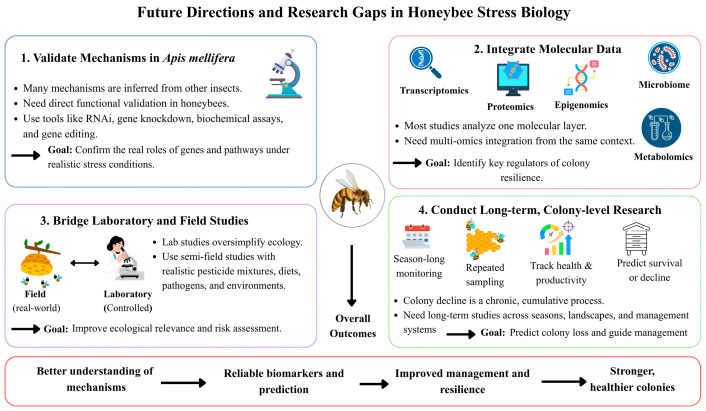
Key research gaps and future directions in honeybee stress biology.

Additionally, long-term colony-level research is still inadequate given the intricacy of the issue. While colony decline frequently develops gradually through chronic sublethal stress, seasonal transitions, and recurrent exposure to interacting pressures, many published investigations concentrate on acute toxicity, short-term physiological responses, or discrete stress events. Colony losses are influenced by complex and cumulative processes rather than one-time shocks, as determined by VanEngelsdorp et al., 2009 [187], and later longitudinal investigations. Similarly, Dainat et al., 2012 [188], demonstrated that colony mortality patterns are best understood across long time periods that capture overwintering, parasite accumulation, food scarcity, and management-related factors. Because of this, even though many short-duration studies are useful mechanistically, they are unable to accurately anticipate whether changes in gene expression, enzyme activity, or microbiome disruption would result in brood loss, queen failure, slower population growth, or final collapse. Therefore, long-term colony monitoring spanning seasons, landscapes, and management systems should be the main focus of future research, with frequent molecular and physiological sampling directly related to survival, productivity, and overwintering success. Long-term research is especially important when there are several stressors. Goulson et al., 2015 [9], showed that low fodder, pesticides, and parasites frequently interact in ways that are only fully apparent over time, when accumulated costs weaken colony resilience. Traynor et al., 2016 [28], stated that managed colonies are frequently exposed to complex pesticide mixes within hive materials, suggesting that repetitive and chronic chemical exposure is more physiologically realistic than acute studies involving a single substance. The idea that robust prediction necessitates integrating environmental history with internal colony biology is further supported by [140], who demonstrated that nutritional stress and landscape context also affect colony condition. Therefore, it will be crucial to conduct long-term investigations that integrate exposure profiling, physiological markers, pathogen dynamics, and colony performance in order to determine which early molecular changes are temporary stress responses and which are accurate indicators of failure. Validating processes in *Apis mellifera*, integrating multi-omics data, coordinating lab and field investigations, and carrying out long-term colony research are all necessary for advancements in honeybee stress biology. By taking these actions, the field will transition from descriptive observations to mechanistic, predictive models of resilience. Key research gaps and priorities for advancing honeybee stress biology are summarized in Figure 3.

This schematic highlights four major priorities: validating mechanisms directly in *Apis mellifera*, integrating multi-omics data, linking laboratory findings with field conditions, and conducting long-term colony-level studies. Together, these approaches will improve mechanistic understanding and enable predictive models of colony resilience and health.

## 10. Conclusions

Honeybee survival relies on a highly integrated defense system in which detoxification, immunity, physiology, behavior, and microbial symbiosis collectively support colony resilience. This review highlights that detoxification in *Apis mellifera* is mediated primarily through core gene families, including cytochrome P450 monooxygenases (CYPs), glutathione S-transferases (GSTs), carboxylesterases (CCEs), ABC transporters, and UDP-glycosyltransferases. These enzymes operate in a tissue-specific manner, with the midgut, fat body, and Malpighian tubules forming the principal detoxification axis. Functional specialization across tissues allows honeybees to metabolize and excrete xenobiotics effectively, while also supporting other physiological processes such as digestion, sensory perception, and oxidative stress management. Innate immune defenses in honeybees comprise multiple signaling pathways, including Toll, Imd, Jak/STAT, JNK, antimicrobial peptide (AMP) production, and RNA interference (RNAi). These pathways function in coordination with tissue-specific effectors such as hemocytes, fat body cells, and gut epithelium, establishing a multi-layered network that balances microbial defense with metabolic and detoxification demands. Importantly, the gut microbiota emerges as a central regulator of both detoxification and immunity, modulating the expression of detoxification genes and AMP synthesis, and influencing physiological resilience under stress conditions. This review emphasizes that honeybee responses are highly plastic and context-dependent. Gene expression is modulated by developmental stage, caste, age, dietary status, and environmental exposures, while epigenetic mechanisms, alternative splicing, and microRNAs provide additional regulatory layers. Heat shock proteins, antioxidant defenses, and proteostasis networks serve as a transversal support system, preserving enzyme functionality and maintaining homeostasis under multi-stressor challenges. These cellular maintenance mechanisms ensure that detoxification and immune pathways can remain operational despite combined exposures to pesticides, pathogens, nutritional limitations, and thermal stress. Multi-stressor interactions are central to colony-level outcomes. Chronic pesticide exposure, pathogen infection, and nutritional stress act synergistically to impair immunity, alter behavior, and reduce overall colony resilience. Experimental evidence demonstrates that sublethal chemical and biological stressors can interact across tissues, affecting detoxification capacity, immune signaling, and neural function, ultimately impacting foraging efficiency, survival, and brood development. The integration of these findings illustrates that honeybee health cannot be understood in isolation, and that predictive assessments require considering the interplay of multiple physiological and environmental factors simultaneously. In summary, the detoxification gene families, immune signaling cascades, organ-specific expression patterns, microbial symbiosis, and multi-stressor interactions collectively define honeybee resilience. These integrated mechanisms provide a foundation for targeted conservation strategies, including selective breeding for stress-tolerant colonies, microbiome-based interventions, and functional validation of candidate genes using molecular tools such as CRISPR. By synthesizing mechanistic insights across detoxification, immunity, and environmental stress, this review presents a comprehensive framework for understanding honeybee health, guiding future research, and informing applied management to safeguard pollinator populations under increasing anthropogenic and climatic pressures.

## Data Availability

No new data were created or analyzed in this study. Data sharing is not applicable to this article.

## Abbreviations

Cytochrome P450 monooxygenases (CYPs), carboxylesterases (COEs), glutathione S-transferases (GSTs), antimicrobial peptides (AMPs), phospholipase A2 (PLA2), Israeli Acute Paralysis Virus (IAPV), deformed wing virus (DWV), American foulbrood (AFB), European foulbrood (EFB), acute bee paralysis virus (ABPV), RNA interference (RNAi), double-stranded RNA (dsRNA), Sacbrood Virus (SBV), small interfering RNAs (siRNAs), Argonaute-2 (Ago2), bee antiviral protein-1 (bap1), intestinal stem cells (ISCs), vitellogenin (Vg), lipopolysaccharides (LPSs), phenoloxidase (PO), lactic acid bacteria (LAB), pattern recognition receptors (PRRs), pathogen-associated molecular patterns (PAMPs), immune deficiency pathway (Imd), long non-coding RNAs (lncRNAs), circular RNAs (circRNAs), naked cuticle gene (nkd), integrated pest management (IPM), reactive oxygen species (ROS), ame-mir-3721-3p microRNA (ame-mir-3721-3p), cytochrome P450 enzymes (P450s), carboxylesterases (CCEs), ATP-binding cassette transporters (ABC), ATP-binding cassette transporter subfamilies A to H (ABCA–ABCH), and 10-formyl tetrahydrofolate dehydrogenase (10-FTHFDH).
